# Conjunctival *FOXP3* Expression in Trachoma: Do Regulatory T Cells Have a Role in Human Ocular Chlamydia trachomatis Infection?

**DOI:** 10.1371/journal.pmed.0030266

**Published:** 2006-08-08

**Authors:** Nkoyo Faal, Robin L Bailey, David Jeffries, Hassan Joof, Isatou Sarr, Mass Laye, David C. W Mabey, Martin J Holland

**Affiliations:** 1 Medical Research Council Laboratories, Fajara, Banjul, Gambia; 2 London School of Hygiene and Tropical Medicine, London, United Kingdom; 3 National Eye Care Programme, Kerewan District Health Centre, Kerewan, Gambia; Moorfields Eye Hospital, United Kingdom

## Abstract

**Background:**

Trachoma, caused by ocular infection with *Chlamydia trachomatis,* remains the leading infectious cause of blindness and in 2002 was responsible for 3.6% of total global blindness. Although transmission can be successfully interrupted using antibiotics and improvements in public and personal hygiene, the long-term success of the control programmes advocated by the World Health Organization are still uncertain. For the complete control and prevention of trachoma, a vaccine would be highly desirable. Currently there are no licensed vaccines for trachoma, and no human vaccine trials have been conducted since the 1960s. A barrier to new attempts to design and introduce a vaccine is the identification of immunologic correlates of protective immunity or immunopathology. We studied important correlates of the immune response in a trachoma-endemic population in order to improve our knowledge of this disease. This is essential for the successful development of a vaccine against both ocular and genital C. trachomatis infection.

**Methods and Findings:**

We used quantitative real-time PCR for C. trachomatis 16S rRNA to identify conjunctival infection. The expression of *IFN-γ, IDO, IL-10,* and *FOXP3* mRNA transcripts was measured. We evaluated the role of immune effector and regulatory responses in the control of chlamydial infection and in the resolution of clinical signs of trachoma in endemic communities in Gambia. All host transcripts examined were detectable even in normal conjunctiva. The levels of these transcripts were increased, compared to normal uninfected conjunctiva, when infection was detected, with or without clinical disease signs. Interestingly, when clinical disease signs were present in the absence of infection, the expression of a regulatory T cell transcription factor, *FOXP3,* remained elevated.

**Conclusions:**

There is evidence of an increase in the magnitude of the local anti-chlamydial cytokine immune responses with age. This increase is coupled to a decline in the prevalence of infection and active trachoma, suggesting that effective adaptive immunity is acquired over a number of years. The anti-chlamydial and inflammatory immune response at the conjunctival surface, which may control chlamydial replication, is closely matched by counter inflammatory or regulatory *IL-10* expression. Differences in the level of *FOXP3* expression in the conjunctiva may indicate a role for regulatory T cells in the resolution of the conjunctival immune response, which is important in protection from immunopathology. However, the expression of cytokines that control chlamydial replication and those that regulate the conjunctival immune response is not simply juxtaposed; the interaction between the infection and the clinical disease process is therefore more complex.

## Abstract 2

The immune response in a trachoma-endemic population showed an increase in local anti-chlamydial cytokine responses with age, associated with a decline in the prevalence of infection and active trachoma.

## Editors' Summary

**Background.**

Trachoma is the leading infectious cause of blindness worldwide. Six million people—most of whom live in crowded, unhygienic conditions with limited water supplies—are blind because of repeated eye infections with Chlamydia trachomatis. This bacterium passes easily from person to person on hands or clothing and is also spread by flies. Successive infections starting in childhood cause progressive scarring of the inside of the eyelid. Eventually, the eyelashes turn inwards and rub painfully over the front of the eye (the cornea). This causes corneal scarring, loss of corneal transparency, and, finally, irreversible loss of sight, usually in adulthood. C. trachomatis infections can be prevented by improving personal hygiene and by reducing fly breeding sites, and they can be treated with antibiotics. In addition, early scarring of the eyelid and turned-in eyelashes can be treated surgically.

**Why Was This Study Done?**

Through the above interventions, the World Health Organization hopes to eliminate trachoma by 2020, but a vaccine might also be necessary. To develop a vaccine, the human immune response to C. trachomatis needs to be better understood. As with other diseases, the immune response to C. trachomatis includes a pro-inflammatory side, which activates immune cells to attack the bacteria, and a regulatory side, which keeps the pro-inflammatory responses in check. The balance between these two sides is not perfect, however. Although the immune response deals with C. trachomatis infections efficiently, it also causes some of the tissue damage that leads to scarring and loss of sight. In this study, the researchers have investigated the human immune response to C. trachomatis to provide immunological information that might help vaccine development.

**What Did the Researchers Do and Find?**

The researchers examined school children living in Gambia, where trachoma is very common, for clinical signs of active trachoma (for example, red or swollen eyelids). To find out which children were infected with *C. trachomatis,* the researchers collected a few cells from the surface of their eyes and looked for a ribonucleic acid (RNA) molecule that is only made by C. trachomatis. The researchers also looked in these samples for human messenger RNA (mRNA) molecules that are made during pro-inflammatory and regulatory immune responses.

The children formed four groups based on infection with C. trachomatis and clinical signs. Some children—particularly the older ones—were uninfected and had no clinical signs. Others were infected but showed no clinical signs—these children were incubating the bacteria. Some were infected and had clinical disease; these children had the highest bacterial loads. Finally, children recovering from an infection carried no bacteria but still had some clinical signs.

The researchers detected different types of immune response in each of these groups. Children incubating the bacteria had a strong pro-inflammatory response—their immune systems were trying to fight off infection. The pro-inflammatory response was even stronger in the infected children with clinical signs, but now the regulatory response had also increased, presumably to limit inflammation. In children in the recovery phase, only regulatory immune cells, which were making an mRNA from a gene called *FOXP3,* remained active.

**What Do These Findings Mean?**

The relative rarity of infections and active disease in older children together with indications of a more active immune response to infection indicates that protective immunity to C. trachomatis is acquired through repeated exposure to it. This bodes well for the development of a vaccine, which would speed up the acquisition of this natural immunity. Furthermore, the new information about immune responses at different stages of infection with C. trachomatis should help in vaccine design. The findings need to be confirmed by tracking immune responses in individual children during episodes of infection, but could then be used to help design vaccines that produce good protective immunity against C. trachomatis without causing too much collateral tissue damage. The current results suggest, for example, that regulatory immune cells are important in limiting the inflammatory response, so vaccine developers may need to ensure that their vaccines stimulate the production of this sort of cell as well as of the pro-inflammatory cells needed to clear the infection.

**Additional Information.**

Please access these Web sites via the online version of this summary at http://dx.doi.org/10.1371/journal.pmed.0030266.

• NHS Direct Online patient information on trachoma

• World Health Organization information on trachoma and its elimination

• US Centers for Disease Control and Prevention general information on trachoma

• MedlinePlus encyclopedia entry on trachoma

## Introduction

Chlamydia trachomatis is the leading infectious cause of blindness and the most common bacterial sexually transmitted infection. In the United Kingdom, the Health Protection Agency found that in 45- to 64-y-old women, rates of chlamydial sexually transmitted infection increased by 177% between 1995 and 2003 [1]. Trachoma, which is caused by repeated ocular infection with *C. trachomatis,* is a progressive disease, occurring in several stages over the lifetime of the individual: active trachoma (follicular conjunctivitis due to C. trachomatis), scarring trachoma, entropion and trichiasis, and eventually blindness due to corneal opacity. The immune response elicited, although important for the control of infection and protection against trachoma [2–4], is also thought be to responsible, at least in part, for the tissue damage that ultimately leads to the scarring sequelae of the disease [5,6].

The processes that lead to the disease and its sequelae are not fully understood. Examination of the immune response as the infection unfolds is key to understanding the immunopathogenesis of ocular C. trachomatis infection (OCI). Infectious diseases are often characterised by an incubation period, in which infection is present without clinical disease; a period during which both infection and disease are present; and a period when clinical signs persist after infection has been cleared [7]. Miller et al. [8] have proposed that OCI follows such a pattern based on the clinical and microbiological observation of trachoma. Earlier work by our group supports this proposal [9]. We identified individuals with infection in the absence of clinical disease, and individuals with clinical signs in the absence of infection. Follow-up examination found that individuals who were PCR-positive but clinically negative were more likely to develop clinical signs than PCR-negative individuals. Conversely, clinical signs were twice as likely to have resolved after 1 mo in PCR-negative individuals with disease as in those who were PCR-positive. Dissection of the immune response in individuals at these times should identify key components of the immune response associated with the acquisition or resolution of infection and clinical signs of trachoma.

Using quantitative PCR it is now possible to study immunity to OCI in humans at the conjunctival surface. This approach has been used in studies in trachoma-endemic populations in Tanzania and Gambia. Bobo et al. [10], in a cross-sectional study of both adults and children, used a polymerase chain reaction–enzyme immuno-assay to detect a number of cytokine transcripts and found a predominantly pro-inflammatory response in trachoma. Burton et al. [11], in a similar study in Gambian individuals, found that transcripts associated with pro-inflammatory, anti-inflammatory, and fibrogenic processes were increased in active trachoma. Markers indicative of a T cell response (interferon gamma [IFN-γ], interleukin [IL]–4, IL-12p40, and perforin) were increased when chlamydial infection was present. This demonstrated that, in a complex response, differences associated with infection and clinical signs could be identified.

A key factor in the immune response to chlamydial infection in both humans and animal models is IFN-γ [12–14]. It has been shown, in tissue culture, to limit tryptophan bioavailability via indoleamine-2,3-dioxygenase (IDO) [15,16]. The action of IFN-γ is often opposed by IL-10, a regulatory cytokine also implicated in chlamydial immunopathogenesis in murine models [17,18]. IL-10 is produced by a variety of cells, but has recently been described as an important effector molecule of some regulatory T cells [19,20]. A role for *Chlamydia*-specific regulatory T cells has not yet been investigated in trachoma; yet evidence from many chronic infectious diseases suggests that these important regulators of the immune response are central to the development of a balanced and effective immune response, particularly at sites of infection. Regulatory T cells can be identified by a combination of phenotypic markers, the majority of which are not unique to this cell type. We used the most reliable marker for naturally occurring and induced regulatory T cells, forkhead box p3 (FOXP3), as an indicator of regulatory T cell activity in different phases of infection and disease caused by OCI.

We present results of a cross-sectional study of OCI and the clinical signs of trachoma in children living in trachoma-endemic communities. The expression of *IFN-γ, IDO, IL-10,* and *FOXP3* in the conjunctiva was investigated using quantitative real-time RT-PCR. The relationship between expression of human mRNAs, the presence and load of bacteria at the conjunctival surface, and clinical signs of active trachoma was examined.

## Methods

### Ethical Approval

The study and its procedures were approved by the Gambia Government/Medical Research Council Joint Ethics Committee and by the Ethics Committee of the London School of Hygiene and Tropical Medicine.

### Recruitment and Follow-Up

School children in nine villages in the Kombo Central and North Bank Divisions of Gambia were examined for the clinical signs of trachoma. A subset of 345 children between the ages of 4 and 15 y were recruited. Children were examined for signs of active trachoma, and a clinical diagnosis made according to the World Health Organization simplified trachoma grading system [21].

Anaesthetic eye drops (Proxymetacaine 0.5%, Minims, Chauvin Pharmaceuticals, Romford, United Kingdom) were administered, and sterile polyester-tipped swabs (Dacron, Hardwood Products Company, Guildford, Maine, United States) were used to obtain cellular material from the upper tarsal conjunctiva of the right eye, by rubbing the swab horizontally four times against the surface of the conjunctiva, turning the swab through 90° each time. Conjunctival swabs were taken for subsequent extraction of RNA; the RNA swab was collected into RNAlater (Ambion [Europe], Huntingdon, United Kingdom), stored on ice, and subsequently stored at −20 °C until RNA was extracted.

Children who were found to have intense inflammatory trachoma (TI) were treated upon diagnosis. After the study each member of the household in which a participant was resident was treated by administration of a single dose of 20 mg/kg oral azithromycin.

### RNA Extraction and RT-PCR

Swabs collected into RNAlater were extracted using the Qiagen RNeasy Kit (Qiagen, Crawley, United Kingdom), according to the manufacturer's protocol, with the following modification: swabs were transferred to a 2-ml screw-cap tube containing RLT buffer, they were vortexed vigorously, and the swab was discarded. A DNase I digestion was carried out during the extraction process to remove contaminating DNA. The RNA was eluted into 50 μl of elution buffer and stored at −20 °C. Twenty-five microlitres of extracted RNA was reverse transcribed using an oligo-dT_15_ primer and the Omniscript reverse transcription system (Qiagen) according to the manufacturer's protocol, and the complementary DNA stored at −20 °C.

### Quantitation of Chlamydial 16S Ribosomal RNA

Three microlitres of total RNA extracted from the ocular swabs was reverse transcribed and amplified by PCR in a one-step reaction using primers specific for chlamydial 16S ribosomal RNA (rRNA). The Quantitect SYBR Green I One-Step RT-PCR kit (Qiagen) was used as described previously [11]. The amount of each chlamydial target per sample was estimated from standard curves generated with each quantitative RT-PCR assay.

### Quantitation of Human mRNA

Total RNA was first reverse transcribed using the Omniscript RT system and 3 μl of the complementary DNA obtained amplified by real-time PCR using primers specific for *IFN-γ, IDO, IL-10, FOXP3,* and *hypoxanthine guanine phosphoribosyl transferase (HPRT)* (Table 1). The copy number of *HPRT* and cytokines per sample were estimated from standard curves included in each assay [11].

**Table 1:**
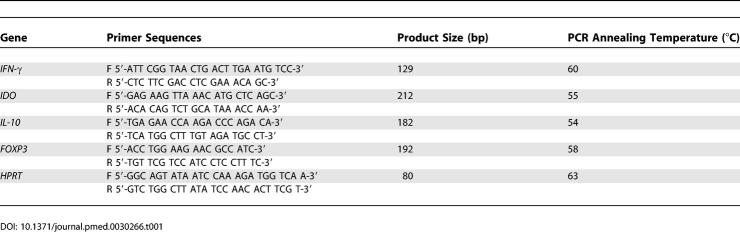
Sequences of Primers Used in Quantitative PCR Assays

### Statistical Analysis

The data were managed using Access (Microsoft, Redmond, Washington, United States) and statistical analyses carried out using Stata 7 (StataCorp, College Station, Texas, United States) and Genstat 8 (VSN International, Herts, United Kingdom). An analysis of variance (ANOVA) with pair-wise comparisons was used for comparisons of expression of chlamydial 16S rRNA across clinical trachoma grades. Individuals were categorised into one of four disease and/or infection states based on the presence of clinical signs of disease and the detection of chlamydial 16S rRNA. We applied the technique of ANOVA as a method to generalise the *t*-test to more than two groups. Group differences were assessed using ANOVA with a covariate adjustment for age. *p*-Values from multiple comparisons were adjusted using the Holm step-down method [22]. Statistical significance was taken to be at the 5% level, but actual *p*-values are reported since these are more informative [23].

Canonical variate analysis was used to demonstrate that the measured cytokine mRNA expression discriminates between the four disease/infection categories [24,25]. Canonical variates are linear combinations of the gene transcript levels that show the most discrimination between the four groups. For each disease/infection group, the mean canonical score and its 95% confidence region can be represented in one or two dimensions, giving a diagrammatic representation of the differences between the groups based on the gene transcript levels. Additionally, examination of the linear combinations may allow the identification of those factors that are most influential in discriminating between the groups.

The Kruskal-Wallis test was used to examine the association between age, OCI and active trachoma. The trend of gene transcript level over age was assessed by allowing different slopes for the age covariate in each of the four categories of disease/infection status. A linear regression model was fitted with terms for age and its interaction with disease/infection status. Increasingly complex models were fitted until there was no improvement in fit at the 5% level.

Correlations in transcript abundance were examined using Spearman's rank correlation coefficient. Trend lines were fitted using locally weighted polynomial regression . Locally weighted polynomial regression is a non-parametric regression technique that is not affected by extreme points.

## Results

### Prevalence of Active Trachoma and Chlamydial Infection

A total of 231 males and 114 females were recruited, with a median age of 8.9 y (4–15 y). The prevalence of active trachoma in either eye (follicular trachoma [TF] and/or TI) was 21.5% (74/345), and the prevalence of scarring trachoma was 5.8% (20/345). The prevalence of chlamydial infection in the right eye (detection of chlamydial 16S rRNA) was 21.8% (72/331). Infection could not be assessed in 14 individuals (ten with normal conjunctiva; four with TF/TI).

**Figure 1:**
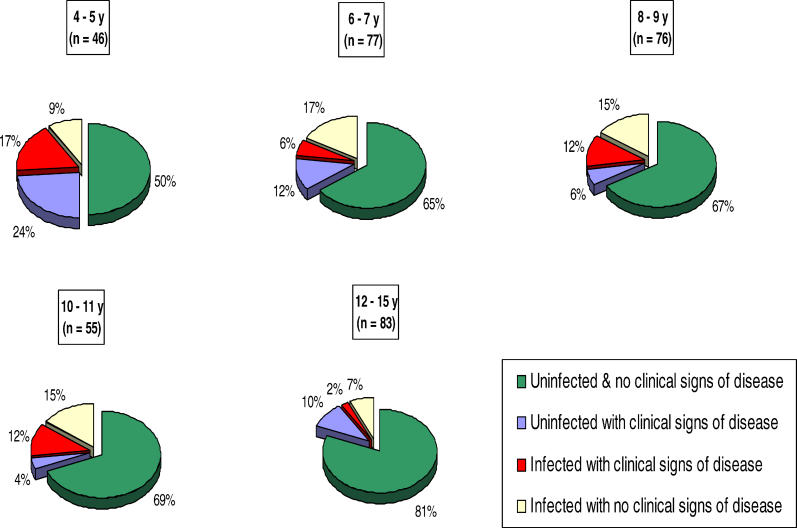
Prevalence of Ocular Chlamydial Infection and Active Trachoma in the Right Eye by Age All children in the cohort were categorised into one of the five age bands. The proportion of children in each of the four disease/infection categories (based on a combination of the presence of clinical signs of active trachoma [TF/TI] and the detection of chlamydial 16S rRNA) is illustrated as pie charts.

In the right eye there was a mismatch between the observation of clinical signs of disease and the detection of infection. Fifty-four percent (35/65) of children with active trachoma (TF and/or TI) did not have evidence of infection, and 58% (42/72) of infected children had no signs of active clinical disease. Significantly higher levels of chlamydial 16S rRNA were detected in infected individuals who also had clinical signs of trachoma (scarring trachoma or active disease) compared to those without clinical signs of disease (Table 2). The pie charts shown in Figure 1 illustrate even over a narrow age range (4–15 y) that the percentage of children with clinical disease (Kruskal-Wallis test: *p =* 0.0005) and infection (Kruskal-Wallis test: *p =* 0.03) is reduced with increased age.

**Table 2:**
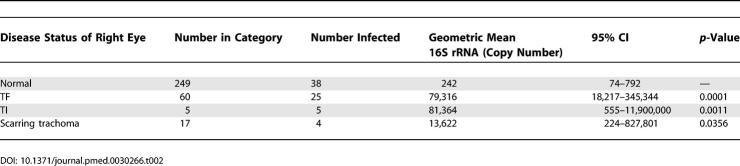
Disease Status of the Right Eye and Geometric Mean Chlamydial 16S rRNA Copy Number from the Right Eye among Infected Individuals Analysed by ANOVA

### Conjunctival Expression of Cytokine Genes and Transcription Factors

**Figure 2:**
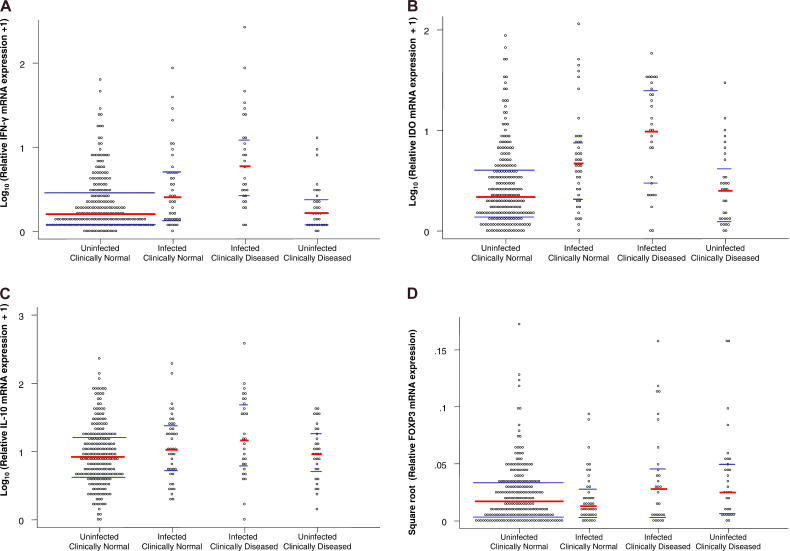
Conjunctival Cytokine mRNA Expression in the Right Conjunctivae of Study Participants RNA was extracted from conjunctival swabs and reverse transcribed, and specific cytokine transcripts were amplified by PCR. Cytokine mRNA expression was normalized against the housekeeping gene *HPRT*. The response of each individual is shown with the median value (red bar) and the 25th and 75th percentiles (blue bars) for each group. Data were tested for significance by ANOVA and adjusted for age. Individuals are categorised using a combination of the presence of clinical signs of active trachoma (TF/TI) and the detection of chlamydial 16S rRNA. *IFN-γ* (A) and *IDO* (B) mRNAs were increased in infected individuals, and they were further increased in those in whom clinical signs coincided with infection compared to clinically normal, uninfected individuals. *IL-10* mRNA (C) was increased only in those who had clinical infections, while *FOXP3* mRNA (D) was increased in both clinically infected and clinically diseased, uninfected individuals.

The medians and inter-quartile ranges of the mRNA transcript ratios for each gene that was tested are shown in Figure 2 for each infection/disease group. To improve the assumptions of ANOVA, the expression ratios for *IL-10*, *IDO,* and *IFN-γ* were log transformed and *FOXP3* was square-root transformed. The overall test by ANOVA for differences between the infection/disease groups, adjusted for age, was significant at the 5% level for each test transcript. The *p*-values for comparisons between the uninfected, clinically normal individuals and all other groups are shown in Table 3. Correction for multiplicity was performed using the Holm step-down method [22], and those values that remained significant at the 5% level are in italics. Because the prevalence of both active clinical disease signs and infection declines with age in trachoma-endemic populations, we adjusted for age in the analysis, i.e., age does not account for the observed statistical differences.

**Table 3:**
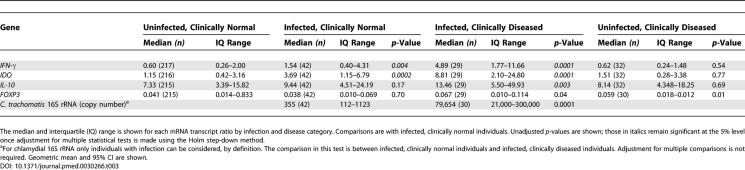
Relative mRNA (Ratio) and Chlamydial 16S rRNA Load from Right Eye Conjunctivae Analysed by ANOVA

Individuals with infection both with and without disease showed significantly higher expression of *IFN-γ* compared to uninfected, clinically normal individuals. There was no significant difference in the level of expression of *IFN-γ* in clinically normal, uninfected individuals compared to those without infection but with clinical disease (Figure 2A). A similar pattern was observed with the expression of *IDO* (Figure 2B).

The expression of *IL-10,* compared to clinically normal, uninfected individuals, was significantly higher only in individuals with infections coincident with clinical signs of disease (*p =* 0.003) (Figure 2C). The expression of *FOXP3* was higher in two groups compared to clinically normal, uninfected children: those in which infection and clinical signs were coincident (*p =* 0.04) and uninfected children with active trachoma (*p =* 0.01) (Figure 2D; Table 3).

**Figure 3:**
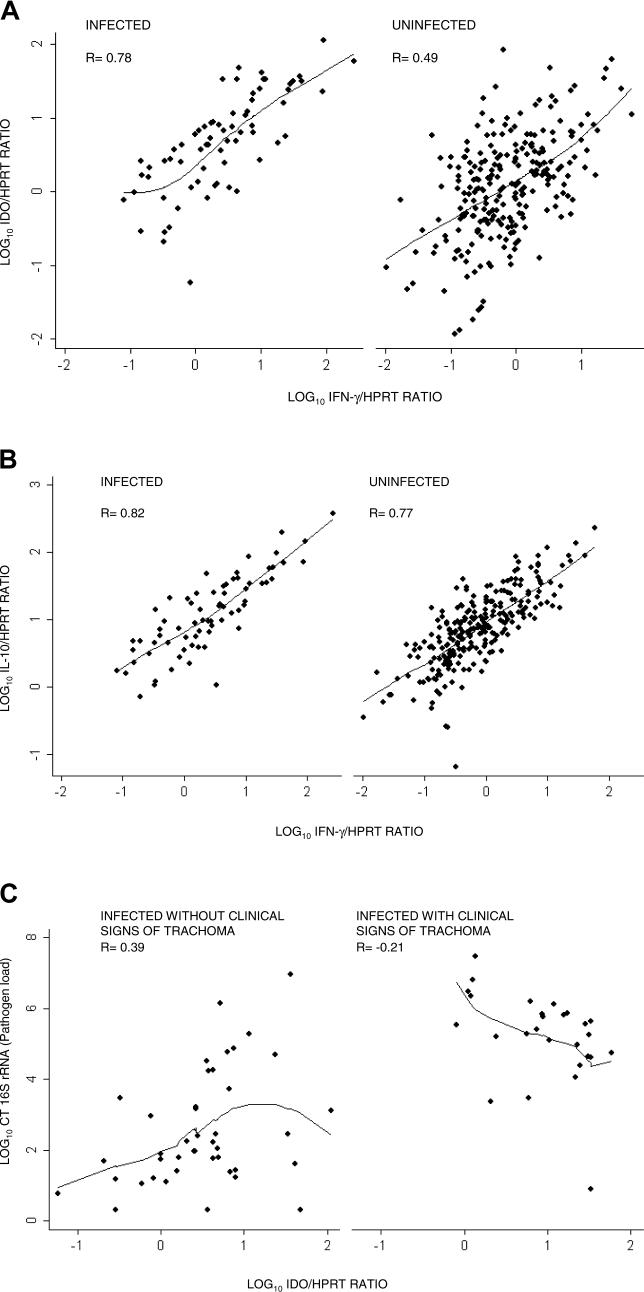
Correlations between Right Eye Conjunctival Gene Transcript Levels Reveal Linked Cytokine Responses and the Relationship to Control of Pathogen Load Abundance of *IFN-γ, IL-10,* and *IDO* relative to *HPRT* expression after log_10_ transformation. Correlation coefficients were calculated by Spearman's rank correlation and trend lines fitted by locally weighted polynomial regression. (A) Strong positive correlation between the conjunctival expression of *IDO* and *IFN-γ* in individuals with infection (*n =* 69) or without infection (*n =* 238). (B) Strong positive correlation between the expression of *IFN-γ* and counter inflammatory *IL-10* in infected (*n =* 69) and uninfected individuals (*n =* 238). (C) Relative abundance of *IDO* and the load of pathogen (chlamydial 16S rRNA) in the conjunctiva. A weak positive correlation was found between pathogen load and *IDO* expression in clinically normal individuals (*n =* 42) (mean pathogen loads are lower in this group; Table 2). In contrast, the relationship between *IDO* expression and pathogen load is weakly negative or absent in individuals with co-incident clinical signs of trachoma (*n* = 29) (pathogen loads are highest in this group; Table 2). CT, *C. trachomatis.*

The expression of *IFN-γ* and *IDO* were positively correlated (Figure 3A) in both infected (*r =* 0.78, 95% confidence interval [CI] 0.67–0.86, *p <* 0.0001) and uninfected individuals (*r =* 0.49, 95% CI 0.39–0.58, *p <* 0.0001). The expression of *IDO* and pathogen load (chlamydial 16S rRNA) had a weak positive correlation (*r =* 0.39, 95% CI 0.09–0.62, *p <* 0.0001) in individuals without clinical signs of disease. However, in individuals with clinical signs there was a weak negative correlation between *IDO* expression and pathogen load (*r = −*0.47, 95% CI −0.72 to −0.11, *p =* 0.014) (Figure 3B). The expression of *IFN-γ* and *IL-10* were also strongly positively correlated, in both infected individuals (*r =* 0.82, 95% CI 0.73–0.89, *p <* 0.0001) and uninfected individuals (*r =* 0.77, 95% CI 0.68–0.80, *p <* 0.0001) (Figure 3C).

### Canonical Variate Analysis of the Expression of Cytokine Genes and Transcription Factors

**Figure 4:**
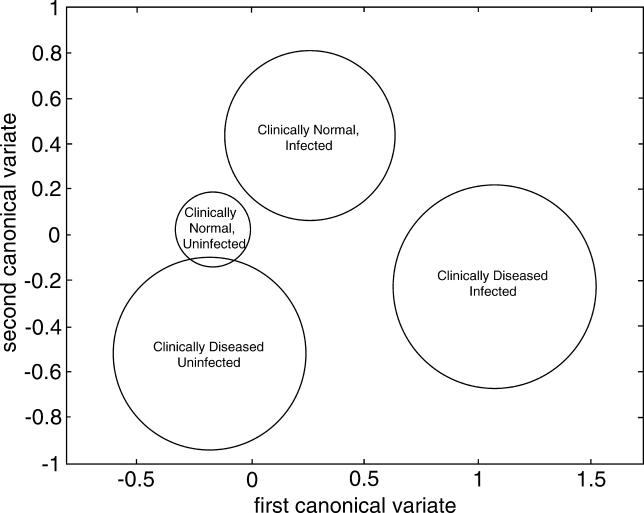
Canonical Variate Analysis Using Right Eye Conjunctival Cytokine mRNA Expression to Distinguish between Individuals Canonical variate analysis was used to form two linear combinations of cytokine mRNA expression that maximized the difference between the disease/infection groups. The means and 95% CIs of these linear combinations have been calculated and plotted in two dimensions for each category, showing that the combinations of cytokine mRNA expression discriminate between the four categories. The canonical variates (CV), with coefficients adjusted to allow for different magnitude scales, are First CV = 0.61*IDO − 0.63*IL-10 + 0.084*FOXP3 + 1.02*IFN-γ Second CV = −0.73*IDO
– 0.43*IL-10 + 1.03*FOXP3 + 0.64*IFN-γ

The first canonical variate (0.61*IDO − 0.63*IL-10 + 0.084*FOXP3 + 1.02*IFN-γ) weights the contribution of each cytokine, where the coefficients have been adjusted to allow for different expression scales. This first canonical variate separates individuals who have clinical signs of disease with coincident infection from all other groups of individuals (Figure 4). In the first canonical variate, *FOXP3* has little influence and the effector cytokines dominate. In the second canonical variate (−0.73*IDO − 0.43*IL-10 + 1.03*FOXP3 + 0.64*IFN-γ), *FOXP3* is the largest contributor, but all cytokines have contributions of similar magnitude. Using both canonical variates separates individuals who are sub-clinically infected (chlamydial 16S rRNA PCR-positive) from those who have clinical signs of active trachoma without infection (Figure 4). These two canonical variates account for 97.3% of the differences between the groups.

### Age-Related Modulation of Cytokine Gene and Transcription Factor Expression: Analysis of Covariance

**Figure 5:**
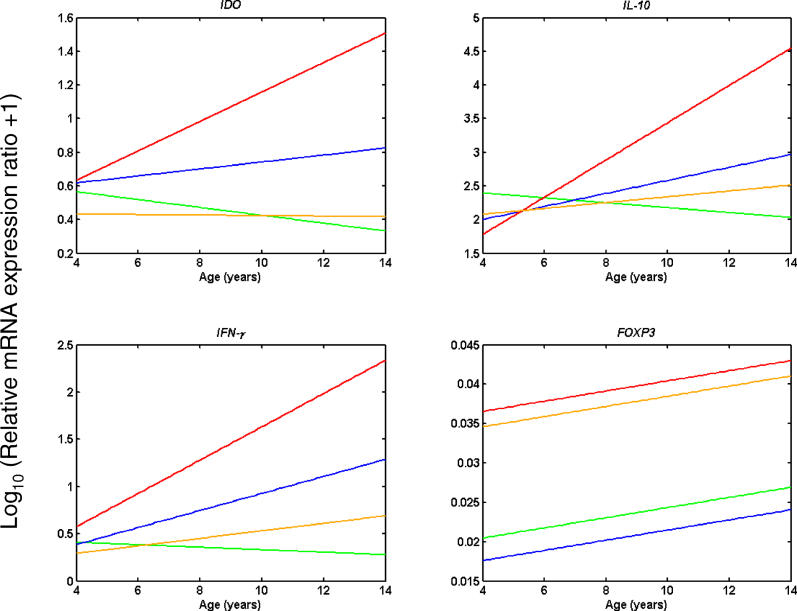
Age Modulation on Right Eye Conjunctival mRNA Expression: Analysis of Covariance Individuals were categorised using a combination of the presence of clinical signs of active trachoma (TF/TI) and the detection of chlamydial 16S rRNA. Regression models were fitted for the expression of each human mRNA assayed and age (years) within each category. The regression lines for individuals in each category are shown for each of the mRNAs, *IDO* (top left), *IFN-γ* (bottom left), *IL-10* (top right), and *FOXP3* (bottom right). Green line: clinically normal, uninfected individuals; blue line: clinically normal, infected individuals; red line: clinically diseased, infected individuals; and orange line: clinically diseased, uninfected individuals.

The amount of variation in gene transcript ratio abundance attributable to the regressions was between 6% *(FOXP3)* and 25% *(IFN- γ).* We looked for evidence of improvement in the magnitude of the immune responses with age and tested whether lines of different slope for age were required within each of the disease/infection strata. Figure 5 shows the most appropriate significant models at the 5% level for each cytokine. Although the regressions were significant, the amount of variation explained was low because age was the only explanatory variable considered. There were significantly different responses with age within the disease/infection groups for all cytokines. Separate lines with significantly different gradients were necessary for *IDO, IL-10,* and *IFN-γ,* in contrast to *FOXP3,* where the separate lines had the same gradient.

The regression model for *FOXP3* shows an increase in *FOXP3* expression with increasing age that is independent of the presence of infection or active trachoma (Figure 5, bottom right). In contrast, the models for *IDO, IFN-γ,* and *IL-10* show no change in gene expression in the “normal” conjunctiva with age. However, there are increases in expression with age that are dependent on the presence of infection and/or disease (Figure 5, top left, top right, and bottom left).

## Discussion

Our data indicate that the greatest chlamydial 16S rRNA gene expression levels are found in those individuals with the highest clinical grade of ocular inflammation (TI). During OCI and the appearance of clinical signs both inflammatory (*IFN-γ*) and regulatory *(IL-10)* cytokine gene expression is increased. Epithelial conjunctival *IDO* expression is also induced, with evidence that the expression levels of these three genes increase with age. The age-related decline in the prevalence of OCI and clinical disease is most likely due to the improvement of these adaptive immune responses. The conjunctival expression of *FOXP3* points to an important contribution of regulatory T cells, which have thus far not been described in chlamydial diseases. Interestingly, of the gene transcripts studied, *FOXP3* is the only transcript expressed above normal levels in individuals with clinical signs of disease but in whom current infection cannot be identified. Further work needs to establish if *FOXP3* expression and regulatory T cells in such individuals are beneficial or exacerbate disease.

Both qualitative DNA-based PCR and quantitative chlamydial 16S rRNA RT-PCR tests identify individuals in whom there is discordance between infection and clinical signs of trachoma. It has been established that the clinical signs of trachoma can persist for long periods of time after infection can no longer be detected [26,27]. Our data are consistent with a classical progression of infection—incubation, concordant clinical signs of disease and infection, followed by the reduction or clearance of infection with residual clinical disease signs—since we find evidence of the existence of these four phases in study participants [8]. We have revealed that it is possible to identify discrete patterns of conjunctival mRNA expression in these phases that are most characteristic of the immune response in each phase. These data provide evidence underscoring the importance of the IFN-γ response in the local control of infection and evidence of the presence of regulatory T cells in human OCI.

In the pre-clinical, incubation phase of infection prior to disease onset there are low chlamydial loads associated with an immune profile that is largely type-1 inflammatory, with increased expression of *IFN-γ* and *IDO*. In a previous study of the conjunctival cytokine responses of a small group of individuals followed over a 6-mo period, we found a strong positive correlation between the amount of chlamydial 16S rRNA and *IFN-γ* mRNA [28]. We now demonstrate that *IDO* is also highly expressed in the conjunctiva. This expression is greatest in the next phase, when clinical signs and the highest infection loads are coincident. Like the expression of pro-inflammatory cytokines, *IL-10* and *FOXP3* expression is also higher in this phase than in normal conjunctiva. The effect of IL-10 and the presence of FOXP3 may possibly counteract the high degree of inflammation; however, antagonism of IFN-γ-mediated chlamydial inhibition by IL-10 may reduce the effectiveness of chlamydial clearance. In humans, host genetic susceptibility to recurrent genital chlamydial infection and to the blinding sequelae of ocular infection (trichiasis) are associated with polymorphism at the *IL-10* locus [29,30]. Interestingly, the strong positive correlation between *IFN-y* expression and *IL-10* in all study participants suggests that the expression of these genes closely moves hand in hand. Similar observations have been made in human leishmaniasis [31], where it was suggested that those with mild forms of cutaneous disease quickly establish parasite-killing mechanisms and efficiently control the inflammatory response, whereas those with disseminated mucosal disease fail to control the inflammatory response [32]. Longitudinal study of individuals with trachoma should reveal if analogous responses contribute to exacerbation of conjunctival infection or effective disease resolution.

These data suggest that the increasing antigen load stimulates higher levels of *IFN-γ/IDO*, which lag behind replication of the pathogen but eventually lead to the control of infection. This is supported by the observation that infected individuals with clinical signs have very high *IDO* transcript levels and relatively lower bacterial loads. An alternative explanation, supported by the generally positive correlation between pathogen load and *IFN-γ/IDO* expression during infection, might be that chlamydial growth evades the IFN-γ/IDO control pathway in the conjunctiva. Currently it is unclear how ocular serovars of *C. trachomatis,* which are deficient in an essential tryptophan synthase gene [33], can withstand the effects of IFN-γ/IDO-mediated tryptophan depletion in conjunctival epithelial cells.

In trachoma-endemic communities, where most individuals are repeatedly exposed to OCI, age is a proxy for antigen experience. In a longitudinal study of individuals of all ages in an endemic community, we found that disease and infection episodes were resolved much more rapidly with increasing age, which explains the observation of a lower prevalence of clinical signs of disease and of C. trachomatis infection in older individuals [34]. The age-related resolution of infection was not explained by age differences in exposure, and presumably indicated the development of an immune response that became more effective at clearing infection with cumulative exposure. The current data demonstrate that, over a narrower age range of individuals (4- to 15-y-olds), a reduction in prevalence of disease and infection can still be observed. In parallel, these findings provide evidence of age-related modulation of cytokine expression in response to disease and infection: older individuals tend to have greater expression of *IFN-γ, IDO,* and *IL-10* in the presence of infection or disease. Thus, it can be suggested that these responses represent components, or markers, of the immune response in the conjunctiva that results in more rapid clearance of infection and disease with increasing age.

In the final phase, when infection has been cleared but signs of active disease persist, there is evidence of the presence of regulatory T cells. Regulatory T cells are known to be involved in the control of a variety of immune responses, including those responses directed against microbial pathogens [35,36]. The mechanisms by which they suppress immune responses have been suggested to include contact-dependent mechanisms, cytokine secretion (in particular IL-10 and TGF-β), and the modulation of antigen-presenting cell function (reviewed in [36,37]). In a murine model of herpetic ocular keratitis, regulatory T cells were able to control the severity of immunopathological lesions in the corneal stroma by reducing migration of pathogenic CD4+ T cells and by reducing the activation of pathogenic T cells, in an IL-10-dependent manner [38].

We found that in OCI, increased *FOXP3* expression at the site of infection could be an indicator of recruitment of regulatory T cells. One of the likely consequences of this recruitment is that the regulatory T cells limit the inflammatory response and the extent of extracellular matrix breakdown (possibly by mechanisms similar to that of the herpetic keratitis model [38,39]), thus limiting tissue damage and encouraging extracellular matrix repair after the damage caused by cellular infiltration and lymphoid follicle formation.

Levels of *FOXP3* were higher in infection and disease, but they also increased with age in both infected and normal conjunctiva. This finding does not indicate that the implied regulatory T cell activity is not C. trachomatis related or disease specific. Rather it may be a feature of the measurement of a transcription factor or an indication that in the conjunctiva the dynamics and magnitude of the adaptive regulatory T cell response are different to that of cytokine gene expression.

There are currently conflicting opinions on whether T cell regulation declines or increases with age, and what contribution this regulation may have to immune senescence or immune dysfunction. In some models of autoimmune disease, onset of auto-reactivity, which is related to age, coincides with the decline of *foxp3*-expressing cells in the tissues [40]. In other diseases, however, such as inflammatory bowel disease, the mucosal lesions contain increased FOXP3+ cells and transcripts even though the compartment of peripheral FOXP3+ cells is reduced [41]. Recently, a subset of cells termed natural naïve FOXP3+ regulatory T cells present in the peripheral blood were also shown to decline with age [40], but others have found that the number of peripheral CD4+CD25^high^ cells with demonstrable regulatory T cell function increased with age [42]. In order to resolve these conflicts, further work is required that clearly identifies the phenotype of each regulatory T cell population and its functional status in relation to its anatomical location.

Studying immune responses at the conjunctival surface in a large number of individuals presents many problems, including limitations in the type of sample that can be obtained. This study used expression of immune response genes to overcome some of these limitations. This is a sensitive technique, amenable to repeated sampling and applicable to large numbers of samples. In addition, it provides greater sensitivity for detecting some of the markers assayed and in some cases is the only means available for detection. However, the nature of the sampling technique precludes an assessment of the relative contribution of different cell types to the production of these cytokines and other factors.

This study highlights the distinct immunological profiles, inflammatory and regulatory, that may be associated with the different phases of OCI and disease. These profiles may, by virtue of their duration, contribute to protection from infection or to the immunopathology induced by infection. Longitudinal follow-up studies should seek to verify their contribution.

## Supporting Information

### Accession Numbers

The National Center for Biotechnology Information (http://www.ncbi.nih.gov) accession numbers for the transcripts used in this study are *FOXP3* (NM_014009), *HRPT* (M31642), *IDO* (M34455), *IFN-γ* (X01992), and *IL-10* (NM_000572).

## Abbreviations

ANOVA: analysis of variance
CI: confidence interval
FOXP3: forkhead box p3
HRPT: hypoxanthine guanine phosphoribosyl transferase
IDO: indoleamine-2,3-dioxygenase
IFN-γ: interferon gamma
IL: interleukin
OCI: ocular *Chlamydia trachomatis* infection
rRNA: ribosomal RNA
TI: follicular trachoma
TI: intense inflammatory trachoma
